# Excitatory and Inhibitory Neurons in the Hippocampus Exhibit Molecularly Distinct Large Dense Core Vesicles

**DOI:** 10.3389/fncel.2016.00202

**Published:** 2016-08-31

**Authors:** José J. Ramírez-Franco, Francisco J. Munoz-Cuevas, Rafael Luján, Sandra Jurado

**Affiliations:** ^1^Department of Pharmacology, University of Maryland School of MedicineBaltimore, MD, USA; ^2^Synaptic Structure Laboratory, Departamento de Ciencias Médicas, Facultad de Medicina, Instituto de Investigación en Discapacidades Neurológicas, Universidad Castilla-La ManchaAlbacete, Spain

**Keywords:** phogrin, hippocampal interneurons, Large Dense Core Vesicles, secretion, vesicle markers

## Abstract

Hippocampal interneurons comprise a diverse family of inhibitory neurons that are critical for detailed information processing. Along with gamma-aminobutyric acid (GABA), interneurons secrete a myriad of neuroactive substances via secretory vesicles but the molecular composition and regulatory mechanisms remain largely unknown. In this study, we have carried out an immunohistofluorescence analysis to describe the molecular content of vesicles in distinct populations of hippocampal neurons. Our results indicate that phogrin, an integral protein of secretory vesicles in neuroendocrine cells, is highly enriched in parvalbumin-positive interneurons. Consistently, immunoelectron microscopy revealed phogrin staining in axon terminals of symmetrical synapses establishing inhibitory contacts with cell bodies of CA1 pyramidal neurons. Furthermore, phogrin is highly expressed in CA3 and dentate gyrus (DG) interneurons which are both positive for PV and neuropeptide Y. Surprisingly, chromogranin B a canonical large dense core vesicle marker, is excluded from inhibitory cells in the hippocampus but highly expressed in excitatory CA3 pyramidal neurons and DG granule cells. Our results provide the first evidence of phogrin expression in hippocampal interneurons and suggest the existence of molecularly distinct populations of secretory vesicles in different types of inhibitory neurons.

## Introduction

GABAergic neurons in the mammalian central nervous system (CNS) comprise a number of interneuronal subgroups that assemble into networks with specific synaptic properties and functional roles (reviewed in Freund and Buzsáki, 1996; McBain and Fisahn, 2001; Klausberger and Somogyi, 2008). A particularly vast heterogeneity of GABAergic cells has been reported in the hippocampal region where different subtypes can de distinguished by their distinct electrophysiological properties and specific neuropeptide content (Freund and Buzsáki, 1996; McBain and Fisahn, 2001; Klausberger and Somogyi, 2008). Recent advances in understanding neuropeptide signaling suggest that the interneuronal system of neuropeptides is crucial for maintaining appropriate brain function. However, it is currently unknown how interneurons regulate neuropeptide storage and release to modulate synaptic transmission and information processing. This lack in our knowledge is in stark contrast to the detailed mechanistic insights into GABA exocytosis (Südhof, 2013). Thus, a better understanding of the role of neuropeptide secretion in different interneuron types is necessary to unveil their function in regulating neuronal networks.

Neuropeptides and non-classical neurotransmitters are generally stored, delivered, and secreted via Large Dense Core Vesicles (LDCVs) which are described as large (70–150 nm) vesicles commonly found at non-specialized release sites (Fried et al., 1985; Zhu et al., 1986). Additionally, neuropeptides can be found in medium sized vesicles (MDCVs; 60–100 nm) of 1/8 of the volume of LDCVs and predominantly located at presynaptic terminals (van den Pol, 2012). Despite the remarkable variability of their cargo, LDCVs and MDCVs have been considered a molecularly homogenous population constituted by common integral proteins which can be found in both neuroendocrine and nerve cells. This is the case of the calcium-activator proteins for secretion (CAPS) and the chromogranin family (Speidel et al., 2003; Machado et al., 2010; Bartolomucci et al., 2011). Chromogranin B (ChB, a.k.a. secretogranin I) expression has been reported in both neuroendocrine cells and neurons (Fischer-Colbrie et al., 1985). Despite these commonalities in molecular composition, different modes of neuropeptide vesicle exocytosis have been reported in cortical and hippocampal neurons where vesicles with distinct cargo undergo either persistent or transient exocytosis (de Wit et al., 2006; Farina et al., 2015).

Consistent with the notion of vesicle heterogeneity in the CNS, the present work has revealed that different hippocampal neurons exhibit distinct LDCV markers. Particularly, we have found that phogrin (a.k.a IA2β and PTPRN2) is highly expressed in parvalbumin positive interneurons (PV^+^). Phogrin is an integral protein of LDCVs in neuroendocrine tissues (Wasmeier and Hutton, 1996) and has been implicated in several roles of the physiology of secretory vesicles including trafficking (Wasmeier et al., 2002, 2005; Saito et al., 2011), exocytosis (Cai et al., 2004), and endocytosis (Torii et al., 2005; Wasmeier et al., 2005). To date phogrin has been widely used as one of the major auto-antigens in diabetes mellitus type I (Kawasaki et al., 1996; Lu et al., 1996) and in the CNS, phogrin expression has been proposed to be developmentally regulated (Chiang and Flanagan, 1996). Although the function of phogrin in nerve cells remains poorly understood, phogrin disruptions have been linked to attention deficits (Lionel et al., 2011), addiction and mood disorders (Yang et al., 2011), Down Syndrome (Papoulidis et al., 2014) and HOXA1 spectrum disorder (Abu-Amero et al., 2012) suggesting a pivotal role in regulating brain development and function.

Here, we have combined electron microscopy, immunohistofluorescence, and transgenic mice to reveal that phogrin is differentially expressed in different subtypes of hippocampal interneurons. Phogrin was found primarily expressed in hippocampal PV^+^ cells but virtually absent from somatostatin (SOM) and cholecystokinin (CCK) interneurons. In addition to being cell specific, phogrin expression was cargo-dependent, as it was also abundant in interneurons containing NPY, especially in the CA3-DG area. Our findings indicate the existence of different populations of secretory vesicles restricted to particular neuronal subtypes, and that phogrin may serve as a novel marker for specific interneuronal subgroups in the hippocampus.

## Materials and methods

### Animals

Adult C5BL/6 wild-type mice were obtained from The Jackson Laboratory. PVCre-Ai6 mice were generated by breeding commercial PV-Cre mice (The Jackson Laboratory, J-008069) with reporter Ai6 mice that express the fluorescent protein ZsGreen1 upon Cre-mediated recombination (The Jackson Laboratory, J-007906). All animals used in this study were males. All animal procedures were performed in accordance of The University of Maryland School of Medicine Institutional Animal Care and Use Committee. The University of Maryland School of Medicine Institutional Animal Care and Use Committee approved the study. All efforts were made to minimize the number of animals used and their suffering throughout the experiments.

### Immunohistofluorescence

Adult (P30–P60) C57BL/6 wild-type mice or PVCre-Ai6 mice were fatally anesthetized and transcardially perfused with ice-cold 4% paraformaldehyde in PBS. Brains were removed and post-fixed overnight (o/n) at 4°C in the same fixative solution. Coronal brain slices of 30 μm were obtained in a KD-400 vibrating microtome (IHC World), collected as floating sections and blocked for 1 h and a half at room temperature (RT) in a solution containing 0.3% Triton X-100 and 5% NGS in PBS. After blocking, sections were incubated (o/n; 4°C) with the following primary anti-bodies in a solution containing 0.3% Triton X-100 and 1% NGS in PBS: Rabbit anti-Phogrin (1:200; Torii et al., 2009); Mouse anti-Phogrin (3 μg/mL; Sigma-Aldrich SAB1406349); Mouse anti-VAMP2 (1:200; Synaptic Systems 103211); Mouse anti-GAD67 (1:1000; Sigma-Aldrich G5419); Rabbit anti-GAD67 (1.5 μg/mL; Boster PA1036); Rabbit anti-NeuN (1:500; Cell Signaling 12943); Rabbit anti-Chromogranin-B (1:200; Synaptic Systems 259103); Rabbit anti-Neuropeptide Y (1:2000; Peninsula laboratories T-4070); Rabbit anti-Somatostatin (1:1000; Peninsula laboratories T-4103); Rabbit anti-Cholecystokinin (1:2000; Sigma-Aldrich C2581). After five washes of 15 min each in PBS containing 0.25% Tween 20, the floating sections were incubated with specific Alexa conjugated antibodies (1:200, Molecular Probes) in a PBS solution containing 0.3% Triton X-100 and 1% NGS (for 2 h at RT. Finally, sections were washed, and incubated with DAPI (Sigma-Aldrich) for 10 min and mounted. Sections were stored at 4°C, and images were acquired on a Zeiss LSM510 Meta confocal scanning microscope.

### Immunohistochemistry for electron microscopy

Electron microscopic examination of immunoreactivity for phogrin in the mouse dorsal hippocampus was performed as described previously using the pre-embedding immunogold method (Luján et al., 1996). Briefly, free-floating sections were incubated in 10% NGS diluted in TBS for 1 h at RT. Sections were then incubated for 48 h in a Mouse anti-Phogrin antibody (Sigma-Aldrich SAB1406349) at a final protein concentration of 1–2 μg/ml diluted in TBS containing 1% NGS. After several washes in TBS, sections were incubated for 3 h in goat anti-mouse IgG coupled to 1.4 nm gold (Nanoprobes Inc., Stony Brook, NY) diluted 1:100 in TBS containing 1% NGS. After several washes in phosphate-buffered saline (PBS), the sections were postfixed in 1% glutaraldehyde diluted in the same buffer for 10 min. They were washed in double distilled water, followed by silver enhancement of the gold particles with a HQ Silver kit (Nanoprobes Inc., Stony Brook, NY). Then, sections were treated with osmium tetraoxide (1% in 0.1 M PB), block-stained with uranyl acetate, dehydrated in graded series of ethanol and flat-embedded on glass slides in Durcupan (Fluka) resin. Regions of interest were cut at 70–90 nm on an ultramicrotome (Reichert Ultracut E, Leica, Austria) and collected on 200-mesh nickel grids. Staining was performed on drops of 1% aqueous uranyl acetate followed by Reynolds's lead citrate. Ultrastructural analyses were performed in a Jeol-1010 electron microscope.

### Measurements and quantification

All quantifications were obtained from a minimum of 10 sections from the dorsal hippocampus per mice. Immunofluorescence was analyzed in the different hippocampal subregions [CA1, CA2, CA3, and dentate gyrus (DG)] using approximately 20–30 fields of view for each condition. Both the granular cell layer and hilus were analyzed together and reported as the DG region. Colocalization of phogrin with NeuN, GAD-67 and interneuron markers was expressed as a percentage of number of positive cells out of phogrin positive cells. Colocalization with VAMP2 and Chromogranin B, and that of GAD67 with Chromogranin B, were expressed as Pearson's correlation coefficient (Adler and Parmryd, 2010). To this end, the Intensity Correlation Analysis plugin in ImageJ (National Institutes of Health) was used (Li et al., 2004), and rolling ball background subtraction was applied to the images before processing. Fields of view containing ventricles or dead pixels were digitally cropped for processing, since it has been reported that this background pixels could yield artifactual results (Nakamura et al., 2007). Absolute percentages were estimated as the fraction of positive cells out of the total number of cells (identified by nuclear DAPI staining) in a given field of view. Normalized percentages were calculated as the absolute percentage of phogrin^+^ cells divided by the absolute percentage of GAD67^+^ cells for each hippocampal subregion. For VAMP2-phogrin and Chromogranin B-phogrin colocalization measurements, phogrin images were thresholded and a binary mask was obtained. This mask was subsequently used to analyze colocalization exclusively in those ROIs corresponding to phogrin positive puncta. When cell densities were expressed as a function of the overall number of cells, the Particle Analysis-Nucleus Counter plugin was employed over the DAPI images corresponding to each field of view; quantifications were then visually surveyed, to assure the proper functioning of this plugin.

### Phogrin antibody validation

The commercially available antibody mouse anti-Phogrin (Sigma-Aldrich SAB1406349) primarily used in this study was validated against the home-made antibody rabbit anti-Phogrin generated by Torii et al. (2009). Double immunohistofluorescence using both antibodies yielded a Pearson's coefficient of 0.84 ± 0.01, indicating a high degree of colabeling of both antibodies in different hippocampal subregions (Supplemental Figures 1, 2) and several other brain regions (Supplemental Figure 3). Pearson's coefficient was calculated using the Intensity Correlation Analysis plugin as described previously in Measurements and quantification.

### Statistics

Data were analyzed using OriginPro 8.0 software. For statistical purposes one-way ANOVA followed by Bonferroni's test was used. Differences were considered statistically significant when *p* < 0.05 with a confidence limit of 95%. All values were expressed as mean ± S.E.M.

## Results

### Interneurons exhibit different vesicle markers than excitatory cells in the hippocampus

**Figure 1:**
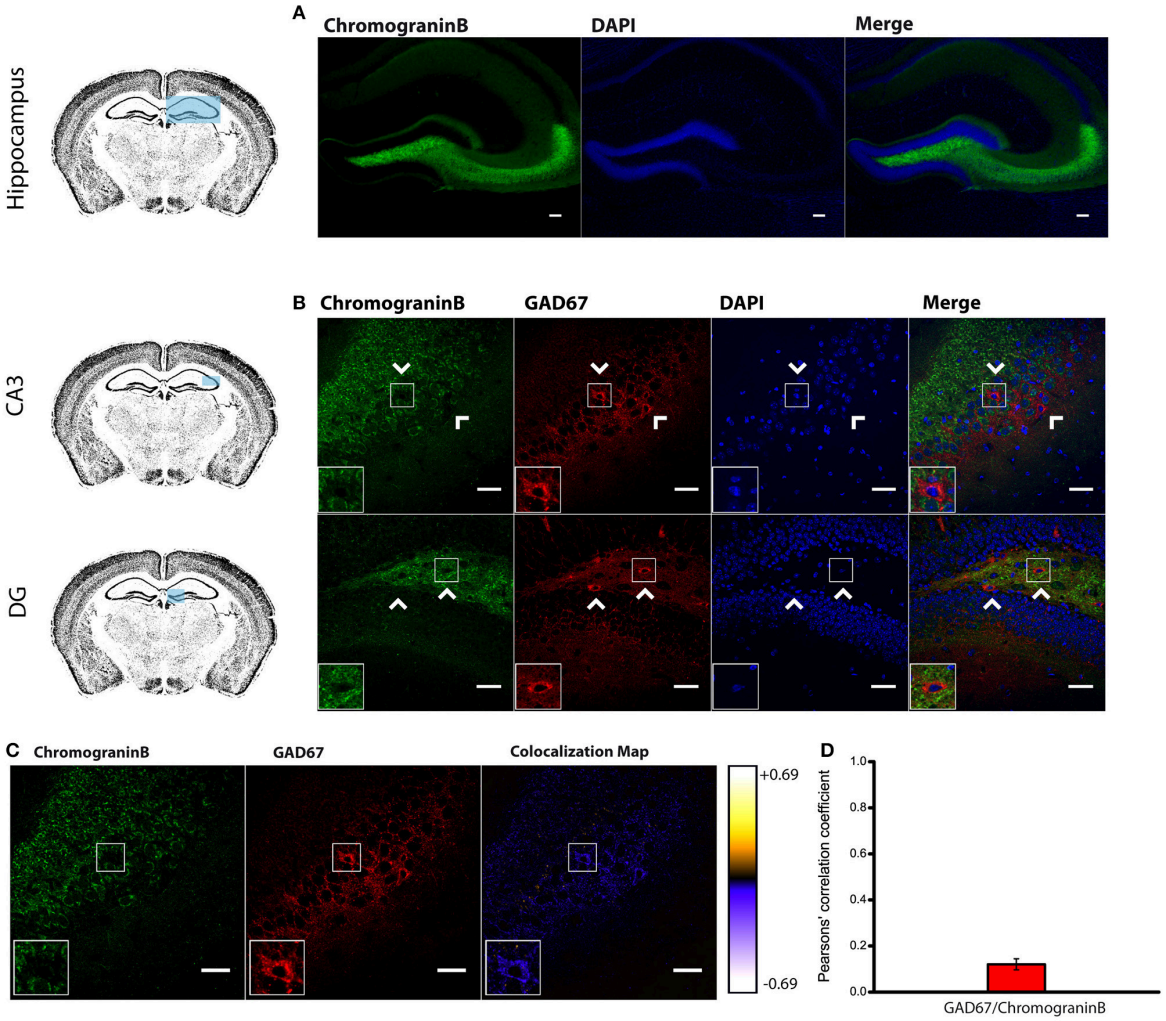
**Endogenous Chromogranin B is not detected in GAD67^**+**^ Cells. (A)** Distribution of endogenous Chromogranin B immunolabeling in the adult mouse hippocampus. Scale bar represents 100 μm. **(B)** Immunohistofluorescence analysis in CA3 and DG of the expression pattern of GAD67 and Chromogranin B showing a marked segregation in hippocampal tissue. Arrow heads indicate Chromogranin B^−^/GAD67^+^ cells. Left-bottom boxes show a magnified view of the area of interest delimited by the smaller box. Scale bar represents 40 μm. **(C)** Chromogranin B and GAD67 labeling and colocalization map obtained from ImageJ Intensity Correlation Analysis plugin. Scale bar represents 40 μm. **(D)** Mean Pearsons' correlation coefficient for GAD67 and Chromogranin B obtained from several CA3 and DG fields (Mean coefficient value = 0.12 ± 0.02; *n* = 48 fields).

To identify the molecular composition of secretory vesicles in different hippocampal interneuron subtypes, we started by characterizing the expression pattern of canonical LDCV markers CAPS-1 and chromogranin B (Speidel et al., 2003; Machado et al., 2010). According to previous work in primary cultured neurons (Farina et al., 2015), immunohistofluorescence analysis in adult mouse slices revealed high levels of CAPS-1 (Supplemental Figure 4) supporting the notion that this protein is associated with neuronal LDCVs. As expected, CAPS-1 was broadly expressed in the hippocampus showing no particular cell-specificity. This was in contrast to chromogranin B staining which was abundant in neurons of the CA3 *stratum pyramidale, stratum lucidum* and in both the hilar region and the molecular layer of the DG (analyzed together as the whole DG region), but mostly absent from the CA1 and CA2 subregions (Figure 1A) consistently with previous reports (Kroesen et al., 1996; Nicolay et al., 2007).

Surprisingly, double labeling of chromogranin B and anti-GAD67 to identify GABAergic neurons (Kanaani et al., 2010) yielded a low mean Pearson's coefficient value (0.12 ± 0.02; *n* = 48 fields; given the low expression levels of chromogranin B in the CA1 and CA2 regions, the mean coefficient value was obtained only from CA3 and DG regions) (Figures 1B–D). Our results indicate that both LDCV-associated proteins CAPS-1 and chromogranin B are not highly expressed in hippocampal interneurons. Particularly chromogranin B, which is primarily absent from GAD67 positive neurons, is unlikely to be an integral component of neuropeptide-containing vesicles in inhibitory cells. These results suggest that interneuronal LDCVs may contain different molecular constituents.

### Phogrin: a vesicle marker of hippocampal interneurons

Given that commonly used LDCVs markers like CAPS-1 and chromogranin B cannot be used to definitely identify secretory vesicles in hippocampal interneurons, we reasoned that interneuronal vesicles may present a distinct molecular composition. We tested phogrin, a transmembrane protein of neuroendocrine vesicles which has been detected during early developmental stages of brain formations that give rise to inhibitory neurons (Chiang and Flanagan, 1996). Because phogrin has been seldom probed in neuronal tissue, we analyzed phogrin expression using electron microscopy and immunohistofluorescence with two different antibodies. Commercially available Mouse anti-Phogrin (Sigma Aldrich SAB1406349) was tested against a Rabbit anti-Phogrin validated by Torii and colleagues for electron microscopy of pancreatic tissue (Torii et al., 2009). As described in Materials and Methods, both antibodies showed nearly identical labeling patterns (Pearson's coefficient of 0.84 ± 0.01), indicating a high degree of co-labeling in the hippocampus (Supplemental Figures 1, 2) and several other brain regions (Supplemental Figure 3).

**Figure 2:**
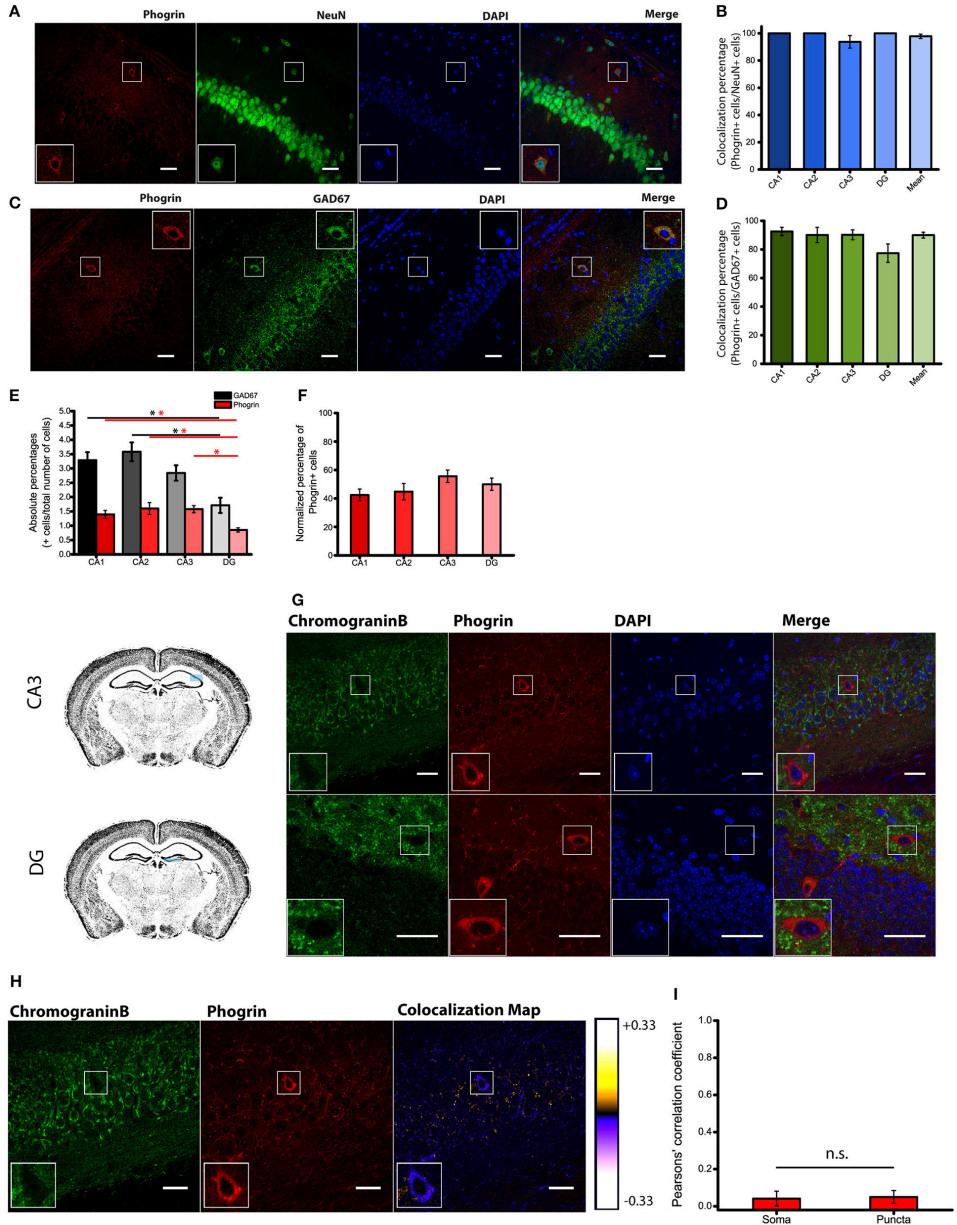
**Phogrin is primarily expressed in interneurons. (A)** Phogrin positive neurons express the pan neuronal marker NeuN. Scale bars represent 40 μm. **(B)** Quantification of NeuN expression in phogrin^+^ cells. Virtually all analyzed phogrin^+^ cells expressed NeuN (91 out of 94 cells). **(C)** Phogrin positive neurons co-express the interneuronal marker GAD67. Scale bars represent 40 μm. **(D)** Quantification of GAD67 expression in phogrin^+^ neurons. The vast majority of phogrin positive neurons were found to be GAD67^+^ (171 out of 197 cells) and no statistical differences were found between different hippocampal subregions. **(E)** Absolute percentages of GAD67 (black and gray bars) and phogrin (red bars) positive cells over the total number of cells measured as the total number of DAPI stained nuclei (624 GAD67^+^ cells and 248 phogrin^+^ cells out of 21,179 cells; ^*^p < 0.05). **(F)** Percentage of phogrin^+^ cells normalized to the percentage of GAD67^+^ cells in each hippocampal subregion. One-way ANOVA followed by Bonferroni's test for means comparison; all the possible comparisons are non-significant. **(G)** Phogrin positive neurons lack Chromogranin B expression in CA3. Scale bar represents 40 μm. Chromogranin B expression is also absent in DG phogrin^+^ neurons. Scale bar represents 20 μm. **(H)** Colocalization map obtained after running Intensity Correlation Analysis Plugin in Image J. **(I)** Mean Pearsons' correlation coefficient for phogrin and Chromogranin B from several CA3 and DG fields (Mean coefficient values: Somas = 0.04 ± 0.04; Puncta = 0.05 ± 0.03; Two sample *t*-test for mean comparison, *p* = 0.87; Somas *n* = 7 fields; Puncta *n* = 11 fields). Boxes within **A,C,G,H** show a magnified view of the area of interest delimited by the smaller box.

Immunohistofluorescence using a commercially available antibody, revealed that phogrin was expressed in discrete cells through all hippocampal regions analyzed (CA1, CA2, CA3 and DG). Phogrin labeling was observed in the *stratum oriens, stratum radiatum* and in the upper layer of *stratum pyramidale* (Supplemental Figure 2). Using double immunohistofluorescence against phogrin and the specific neuronal marker NeuN we confirmed that phogrin expression was limited to neurons in the different hippocampal subregions (colocalization percentages: CA1: 100 ± 0%; CA2: 100 ± 0%; CA3: 93.75 ± 4.58%; DG: 100 ± 0%; 91 NeuN^+^ cells out of 94 phogrin^+^ cells; Figures 2A,B). The morphology and localization of phogrin^+^ neurons suggested that phogrin may be primarily expressed in interneurons. To confirm this possibility we performed double immunohistofluorescence against phogrin and the GABAergic neuron marker GAD67. Surprisingly, the vast majority of phogrin-expressing neurons were GAD67 positive cells suggesting phogrin is a specific marker for interneuronal vesicles in the hippocampus (Normalized percentage as a fraction of GAD67^+^ cells: CA1: 92.54 ± 2.86%; CA2: 90.10 ± 5.34%; CA3: 90.20 ± 3.45%; DG: 77.38 ± 6.46%; 171 GAD67^+^ cells out of 197 phogrin^+^ cells; Figures 2C,D).

Since most of phogrin^+^ neurons were also GAD67^+^ cells, we quantified the density of phogrin^+^ neurons as a fraction of the densities of GAD67^+^ cells. We found no differences between the densities of phogrin^+^ cells expressed as a percentage of GAD67^+^ cells throughout the different hippocampal regions (colocalization percentages: CA1: 42.40 ± 4.15%; CA2: 44.79 ± 5.76%; CA3; 55.58 ± 4.38%; DG: 49.96 ± 4.30%; Figures 2E,F). Our data indicate that contrary to previous suggestions (Chiang and Flanagan, 1996), phogrin expression is abundant in the adult hippocampus and other brain regions (Supplemental Figures 2, 3). More importantly, phogrin is selectively expressed in inhibitory neurons which is suggestive of a specific role in the regulation of interneuronal vesicles in the postnatal hippocampus.

### Phogrin does not colocalize with the canonical LDCVs marker chromogranin B

Consistent with selective labeling of phogrin in hippocampal interneurons, we found a completely separation in the expression pattern of phogrin and chromogranin B in both puncta and somatic regions (Figures 2G–I) (Mean Pearsons' coefficient value: Somas = 0.04 ± 0.04; Puncta = 0.05 ± 0.03). Mean Pearsons' coefficient value was calculated in both CA3 and DG since chromogranin B was undetectable in CA1 and CA2 regions (Figure 1A). These results suggest that phogrin and chromogranin B are segregated in unique subpopulations of vesicles specific to inhibitory or excitatory hippocampal neurons, respectively.

### Subcellular expression of phogrin in hippocampal interneurons

**Figure 3:**
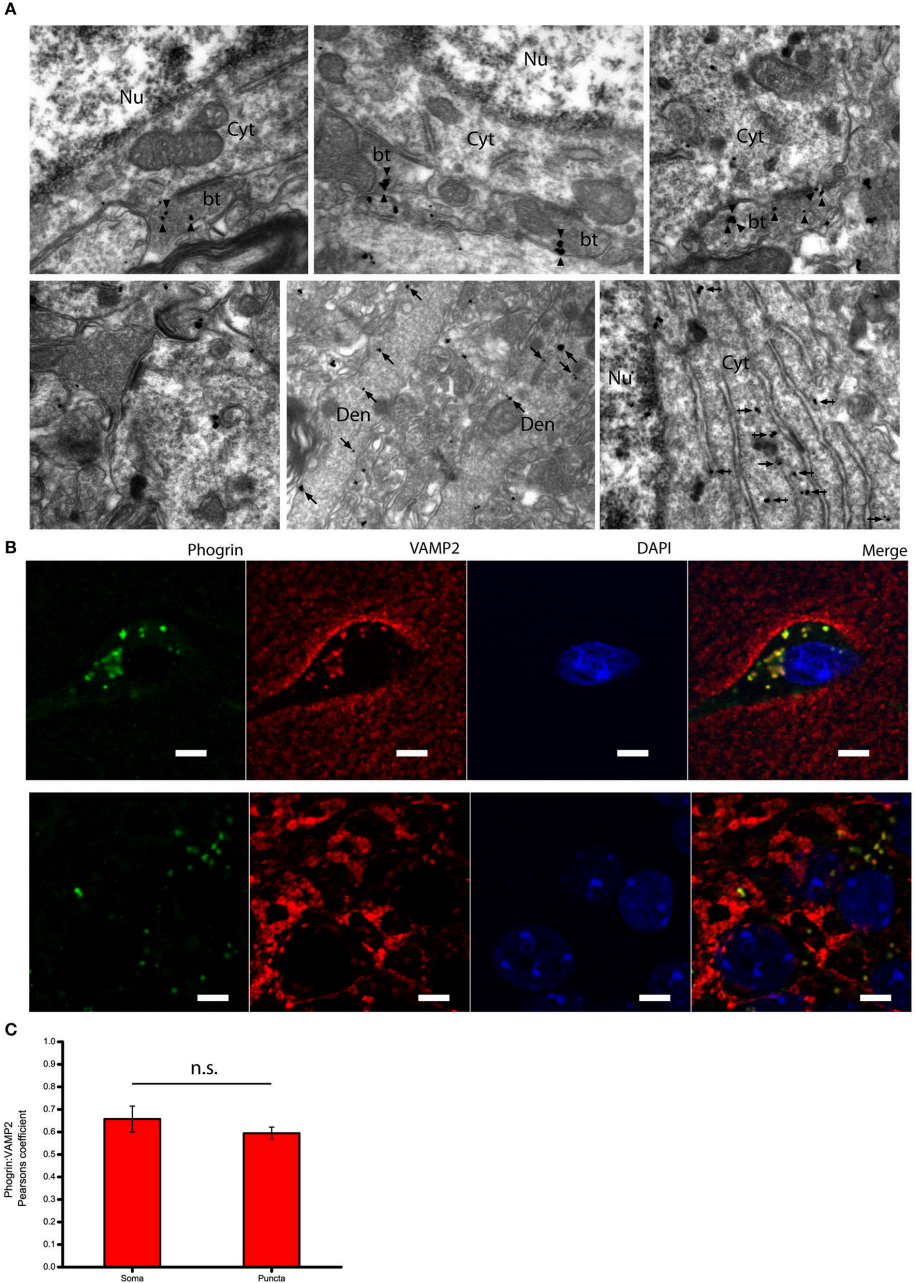
**Subcellular expression of phogrin in hippocampal interneurons. (A)** Immunoreactivity for phogrin in the CA1 region of the hippocampus as revealed using a pre-embedding immunogold method. Immunoparticles for phogrin (black dots) were mainly located at presynaptic sites in button terminals (bt) opposing the cell body of pyramidal cells (arrow heads). Immunoparticles for phogrin were never detected in axon terminals of excitatory synapses recognized by the presence of a prominent postsynaptic density. A significant proportion of immunoparticles for phogrin (arrows) were also detected at postsynaptic sites in dendritic shafts (Den) of interneurons, as well as associated with the rough endoplasmic reticulum (large arrow heads). Cyt, cytoplasm; Nu, nucleus. Scale bars represent 0.5 μm. **(B)** Endogenous phogrin is co-expressed with VAMP2 in cell somas of *stratum oriens* and in puncta in *stratum pyramidale* in axo-somatic contacts. Scale Bar represents 5 μm. **(C)** Pearsons' correlation coefficients for VAMP2 and phogrin colocalization in both somas of *stratum oriens* and puncta in *stratum pyramidale*. (Mean coefficient values: Somas = 0.70 ± 0.03; Puncta = 0.63 ± 0.02. Two sample *t*-test for mean comparison, *p* = 0.18; Somas *n* = 4 fields; Puncta *n* = 14 fields).

Given that the subcellular expression of phogrin in the postnatal brain has been seldom explored, we performed immunolabeling using a pre-embedding immunogold method in the CA1 region of the hippocampus (Figure 3A). Immunoparticles for phogrin were mainly located at presynaptic sites in axon terminals of basket cells establishing inhibitory synaptic contacts with the cell body of pyramidal cells. Furthermore, phogrin labeling was never detected in axon terminals establishing excitatory synapses recognized by the presence of a prominent postsynaptic density in the postsynaptic element. A significant proportion of immunoparticles for phogrin were also detected at postsynaptic sites in dendritic shafts of interneurons, as well as associated with the rough endoplasmic reticulum in the cell body of interneurons. We expanded our analysis by performing double immunofluorescence against phogrin and the synaptic vesicle protein VAMP2. VAMP2 (a.k.a synaptobrevin-2) is an integral component of the SNARE complex required to exocytose vesicles thus can be used to detect presynaptic localizations enriched with fast neurotransmitter-containing vesicles (Südhof, 2013). Additionally, VAMP2 can also be part of postsynaptic membrane compartments where can mediate activity-dependent exocytic events at dendrites (Jurado et al., 2013; Jurado, 2014). Both VAMP2 and phogrin showed a punctuated labeling typical of membrane associated-proteins (Figure 3B). Agreeing with previous findings in rat primary hippocampal neurons (Jiang et al., 1998), VAMP2 and phogrin exhibited a high degree of colocalization in punctate regions but also in the somas (Figure 3C; Mean Pearsons' coefficient values: Somas = 0.70 ± 0.03; Puncta = 0.63 ± 0.02). The subcellular expression of phogrin-containing vesicles points to the possibility that these vesicles may coexist with small synaptic vesicles at presynaptic sites but also be stored at non-canonical sites like postsynaptic localizations.

### Phogrin is a selective marker of PV and NPY interneurons

**Figure 4:**
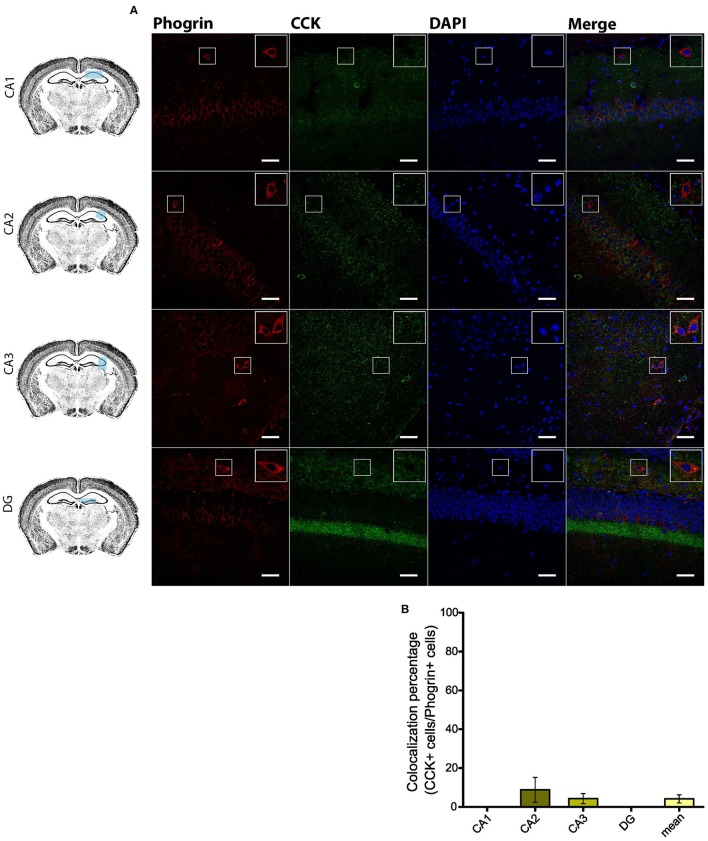
**Phogrin does not colocalize with CCK-positive interneurons in the hippocampus. (A)** Phogrin is not co-expressed with CCK in CA1, CA2, CA3, or DG. Rigth-top boxes within each panel show a magnified view of the area of interest delimited by the smaller box. Scale bars represent 40 μm. **(B)** Colocalization percentage of phogrin^+^ and CCK^+^ interneurons throughout different hippocampal subregions (5 CCK^+^ cells out of 132 phogrin^+^ cells). Non-significant statistical differences were found between subregions. One-way ANOVA followed by Bonferroni's test for means comparison; all the possible comparisons are non-significant.

**Figure 5:**
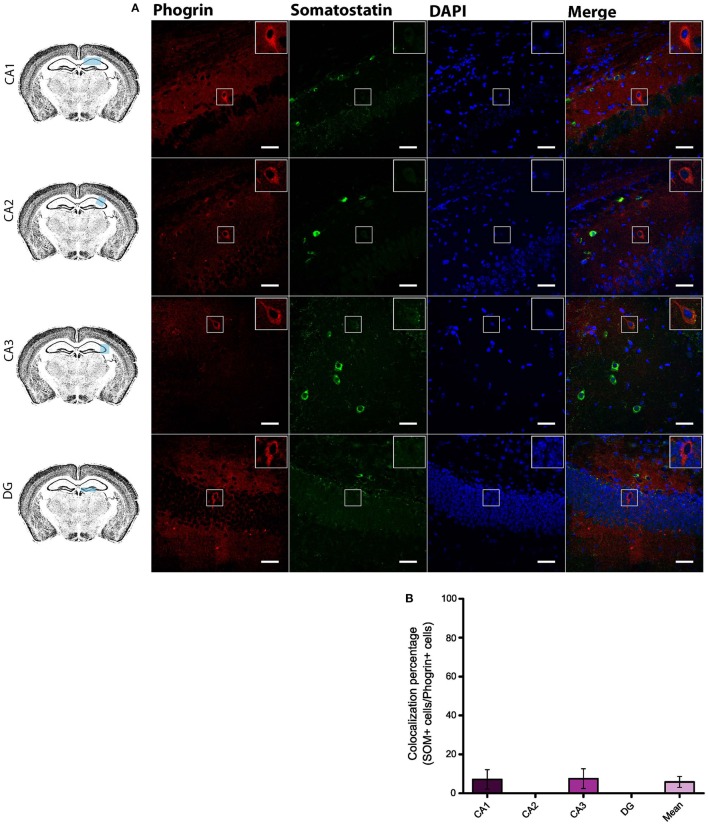
**Phogrin does not Colocalize with SOM-positive Interneurons in the Hippocampus. (A)** Phogrin is not co-expressed with SOM in CA1, CA2, CA3, or DG. Scale bars represent 40 μm. Rigth-top boxes within each panel show a magnified view of the area of interest delimited by the smaller box. **(B)** Colocalization percentage of phogrin^+^ and SOM^+^ interneurons throughout different hippocampal subregions (6 CCK^+^ cells out of 49 phogrin^+^ cells). Non-significant statistical differences were found between subregions. One-way ANOVA followed by Bonferroni's test for means comparison; all the possible comparisons are non-significant.

Given the prevalent expression of phogrin in GAD67^+^ neurons, we sought to identify which interneuron subtypes may selectively exhibit this newly characterized vesicle marker. Firstly, we carried out double staining between phogrin and CCK-containing interneurons (CCK), a subclass of basket cell interneurons which almost exclusively project to the *stratum pyramidale* (Klausberger and Somogyi, 2008). Strikingly, CCK^+^ interneurons showed a total absence of phogrin in all hippocampal subregions analyzed (Figure 4; percentage of CCK^+^ cells out of phogrin^+^ cells: CA1: 0.00 ± 0.00%; CA2: 8.82 ± 6.41%; CA3: 4.32 ± 2.60%; DG: 0.00 ± 0.00%; 5 CCK^+^ cells out of 132 phogrin^+^ cells). Extended characterization to somatostatin positive (SOM^+^) interneurons, another prominent class of basket cells in the hippocampus, revealed almost identical phogrin exclusion (Figure 5; percentage of SOM^+^ cells out of phogrin^+^ cells: CA1: 7.14 ± 5.00%; CA2: 0.00 ± 0.00%; CA3: 7.50 ± 5.09%; DG: 0.00 ± 0.00%; 6 SOM^+^ cells out of 49 phogrin^+^ cells). These results indicate that phogrin expression is restricted to subpopulations of hippocampal interneurons.

**Figure 6:**
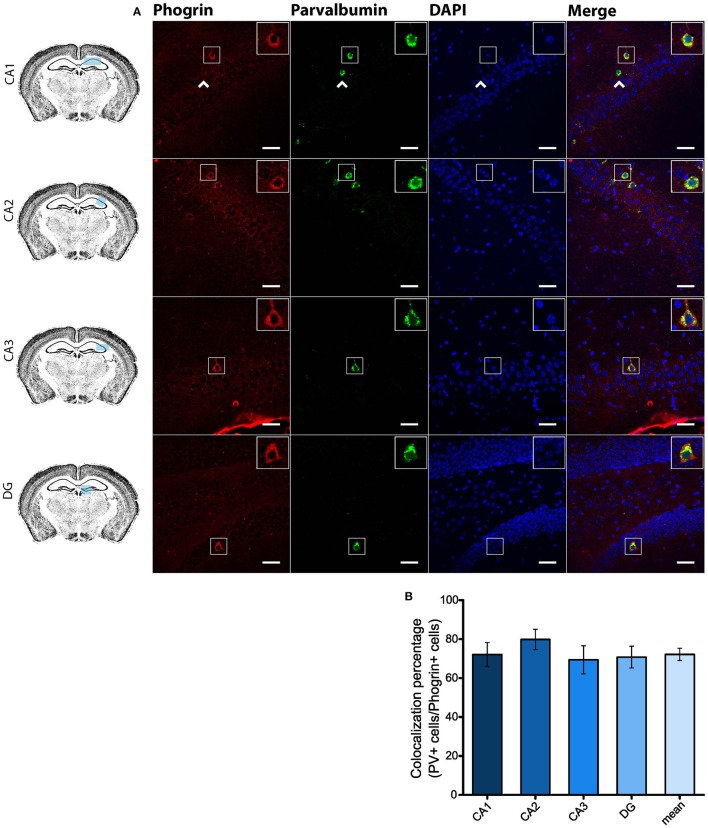
**Phogrin is highly expressed in PV-positive interneurons in the hippocampus. (A)** Phogrin is co-expressed with PV in CA1, CA2, CA3, or DG. Arrow heads indicate phogrin^−^/PV^+^ interneurons. Rigth-top boxes within each panel show a magnified view of the area of interest delimited by the smaller box. Scale bars indicate 40 μm. **(B)** Colocalization percentage of phogrin^+^ and PV^+^ interneurons throughout different hippocampal subregions (225 PV^+^ cells out of 311 phogrin^+^ cells; *n* = 49 fields). Non-significant statistical differences were found between subregions. One-way ANOVA followed by Bonferroni's test for means comparison; all the possible comparisons are non-significant.

**Figure 7:**
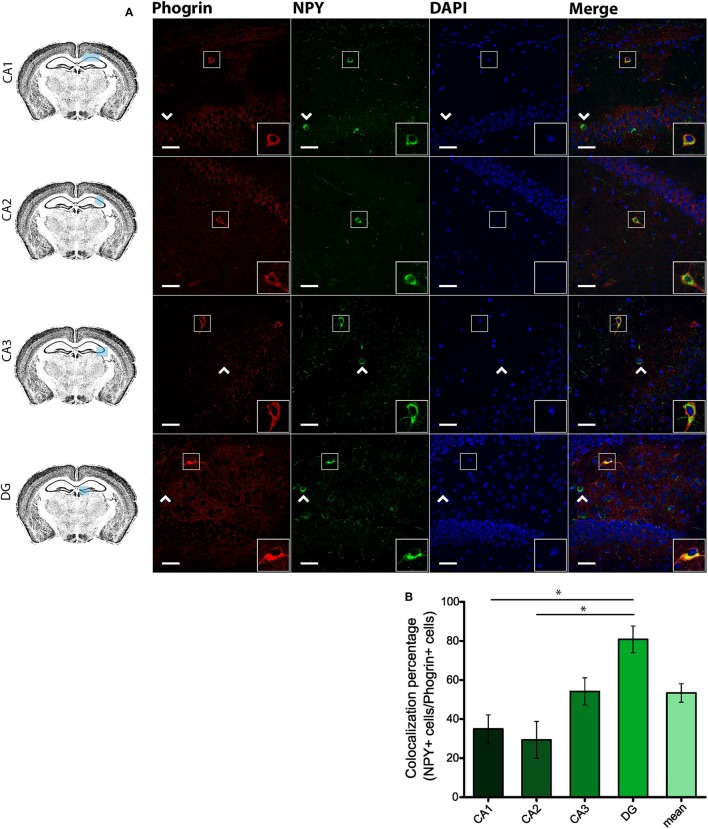
**Phogrin and NPY show a region-dependent pattern of colocalization. (A)** Phogrin is partially co-expresssed with NPY in CA1 and CA2 in contrast to the high degree of colocalization in CA3 and DG. Arrow heads indicate phogrin^−^/NPY^+^ interneurons. Rigth-bottom boxes within each panel show a magnified view of the area of interest delimited by the smaller box. Scale bars represent 40 μm. **(B)** Colocalization percentage of phogrin^+^ and NPY^+^ interneurons throughout different hippocampal subregions (73 NPY^+^ cells out of 156 phogrin^+^ cells; ^*^p < 0.05). One way ANOVA followed by Bonferroni's test for means comparison; non-statistical significant comparisons are not indicated in the graph for clarity.

**Figure 8:**
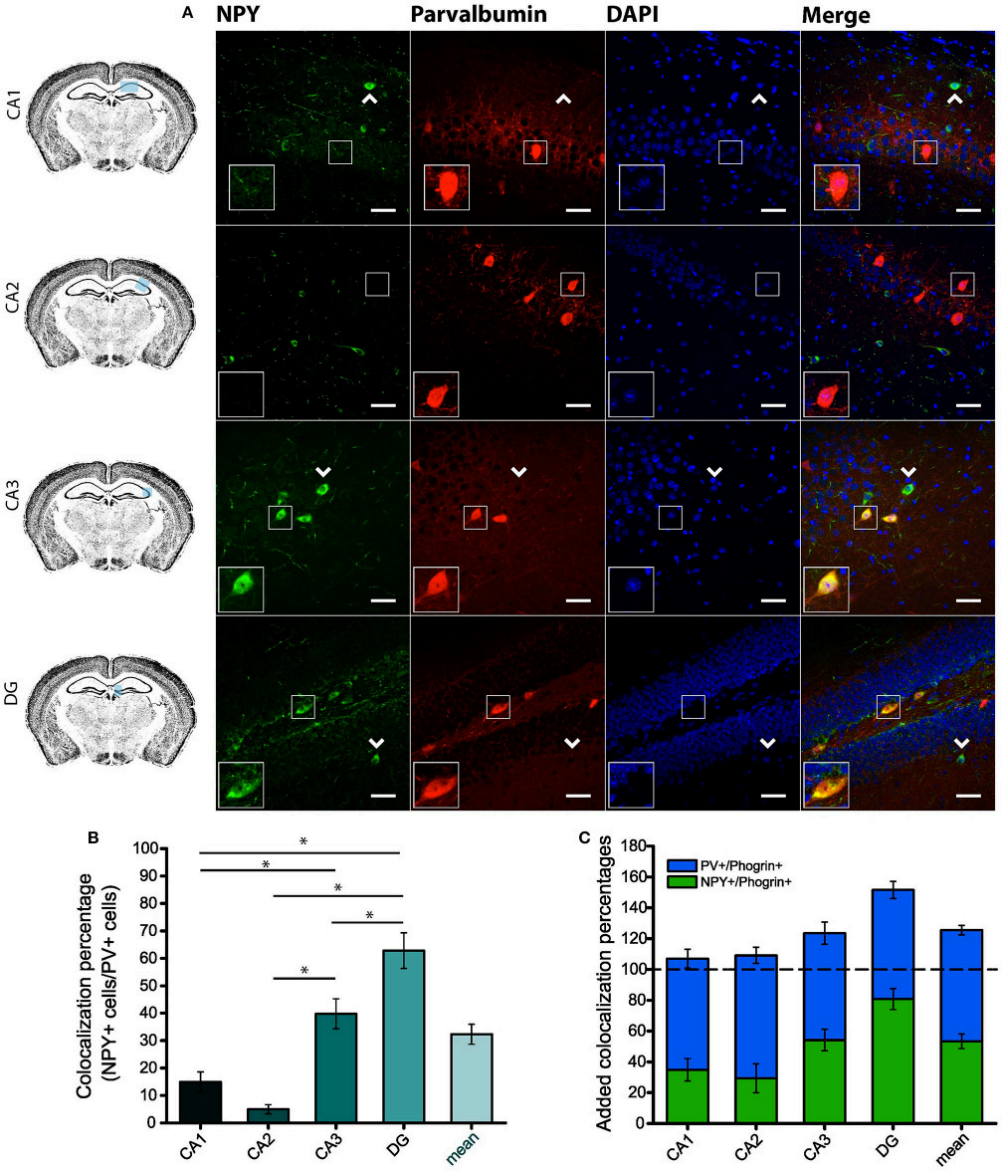
**A specific subpopulation of PV^**+**^ interneurons also contains NPY. (A)** NPY is partially co-expresssed with PV in CA3 and DG but not in CA1 and CA2 subregions. Arrow heads indicate PV^−^/NPY^+^ interneurons. Left-bottom boxes within each panel show a magnified view of the area of interest delimited by the smaller box. Scale bars represent 40 μm. **(B)** Colocalization percentages of NPY and PV in different hippocampal subregions (99 NPY^+^ cells out of 393 PV^+^ cells; ^*^p < 0.05). **(C)** Bar graph represents overlapping mean colocalization percentages of phogrin^+^/NPY^+^ and PV^+^/NPY^+^ interneurons throughout different hippocampal subregions. Note that the value is greater than 100% in CA3 and DG. One way ANOVA followed by Bonferroni's test for means comparison; non-statistical significant comparisons are not indicated in the graph for clarity.

Since GAD67 expression is preferentially associated to PV^+^ interneurons (Fukuda et al., 1997), we hypothesized that phogrin may be restricted to this interneuronal population. To better address this possibility, we performed single immunohistofluorescence against phogrin in PVCre-Ai6 mice to identify PV-containing neurons. Consistently, we found that most of the phogrin-expressing interneurons were PV^+^ in all the hippocampal regions examined (Figure 6; percentage of PV^+^ cells out of phogrin^+^ cells: CA1: 72.08 ± 6.17%; CA2: 79.79 ± 5.25%; CA3: 69.36 ± 7.24%; DG: 70.73 ± 5.56%; 225 PV^+^ cells out of 311 phogrin^+^ cells). Intriguingly, phogrin did not colocalize with PV^+^ interneurons in cortical areas (Supplemental Figure 5) suggesting that phogrin presence in these interneuronal subtype is region-specific. Lastly, we explored the colocalization of phogrin with NPY, another prominent peptidergic marker of hippocampal interneurons (Freund and Buzsáki, 1996). We also observed strong colocalization between phogrin and NPY-expressing cells (Figure 7). Interestingly, the colocalization percentage of phogrin and NPY showed a gradual increase along different hippocampal subregions with the highest level of colocalization in the DG region (Figure 7; percentage of NPY^+^ cells out of phogrin^+^ cells: CA1: 34.91 ± 7.24%; CA2: 29.33 ± 9.43%; CA3: 54.16 ± 6.94%; DG: 80.83 ± 6.81%; 73 NPY^+^ cells out of 156 phogrin^+^ cells). Since we have found that phogrin expression is fairly homogenous throughout the different hippocampal subregions (Supplemental Figure 2), the increase in the colocalization ratio between phogrin and NPY may be likely due to higher levels of NPY expression in the DG (Deller and Leranth, 1990; Gruber et al., 1994). Finally, we tested the percentage of NPY^+^ cells that also were PV^+^. As expected, the degree of colocalization was high (Figure 8A) and increased following the same pattern of phogrin and NPY co-expression (Figure 8B; Percentage of NPY^+^ cells out of PV^+^ cells: CA1: 14.93 ± 3.64%; CA2: 4.95 ± 1.70%; CA3: 39.75 ± 5.45%; DG: 62.78 ± 6.50%; 99 NPY^+^ cells out of 393 PV^+^ cells). Since PV^+^ and NPY^+^ interneurons are thought to belong to unique interneuronal populations, the addition of the colocalization percentages of PV^+^ cells / Phogrin^+^ cells and NPY^+^ cells / Phogrin^+^ cells was expected to be 100% maximum. Surprisingly, added percentages in CA3 and DG subregions were higher than 100% (Figure 8C) suggesting that a significant percentage of interneurons in these hippocampal regions contain both markers. Our findings indicate that phogrin is differentially expressed in a specific subgroup of basket cells which suggest a distinct molecular composition of neurosecretory vesicles in peptidergic hippocampal interneurons. Given its high specificity, phogrin can be used as a novel marker to identify PV-containing neurons in the whole hippocampus and NPY-containing PV^+^ cells in the CA3 and DG subregions.

## Discussion

Interneurons are often classified according to neuropeptide content which usually concurs with distinct electrophysiological properties (reviewed in Freund and Buzsáki, 1996; McBain and Fisahn, 2001; Klausberger and Somogyi, 2008). However, it is becoming increasingly clear that interneuronal neuropeptides are more than convenient neurochemical markers and can act as important modulators of neuronal activity (Baraban and Tallent, 2004). In particular, interneuronal neuropeptides appear to play roles in cognition (Dutar et al., 2002) and as endogenous anti-epileptic agents (Erickson et al., 1996). Although neuropeptide release underlies the integrative role of interneurons, it is still unclear how specific neuropeptide content mediates interneuron function. In answering this question, it is important to elucidate the mechanisms of neuropeptide storage and secretion in distinct interneuronal subtypes.

Neuropeptides are confined to specialized vesicles (LDCVs and MDCVs) which are often released at non-canonical release sites in a slower manner than traditional neurotransmitters (Fried et al., 1985; Zhu et al., 1986; van den Pol, 2012). Most of our knowledge of the molecular composition and exocytosis of secretory vesicles comes from studies in neuroendocrine tissue where a number of markers have been identified. Thus, the chromogranin family (a.k.a. secretogranins) and CAPS have been found in both endocrine and neuronal tissue (Speidel et al., 2003; Machado et al., 2010; Bartolomucci et al., 2011). These findings pointed to the notion that despite the striking diversity of cargo, neuronal secretory vesicles may exhibit a fairly homogenous molecular composition. In contrast to this convention, our data demonstrates that different interneuronal types exhibit distinct vesicle markers (for a summary see Table 1).

**Table 1:** **Summary of colocalization percentage and Pearson's correlation coefficient for different markers used in this study**.

**Colocalization method**	** 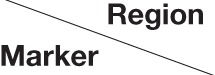 **	**CA1**	**CA2**	**CA3**	**DG**
Colocalization percentage	NeuN:Phogrin	100 ± 0.00%	100 ± 0.00%	93.75 ± 4.58%	100 ± 0.00%
	GAD67:Phogrin	92.54 ± 2.86%	90.10 ± 5.34%	90.20 ± 3.45%	77.38 ± 6.46%
	CCK:Phogrin	0.00 ± 0.00%	8.82 ± 6.41%	4.32 ± 2.60%	0.00 ± 0.00%
	SOM:Phogrin	7.14 ± 5.00%	0.00 ± 0.00%	7.50 ± 5.09%	0.00 ± 0.00%
	PV:Phogrin	72.08 ± 6.17%	79.79 ± 5.25%	69.36 ± 7.24%	70.36 ± 5.56%
	NPY:Phogrin	34.91 ± 7.24%	29.33 ± 9.43%	54.16 ± 6.94%	80.83 ± 6.81
	NPY:PV	14.93 ± 3.64%	4.95 ± 1.70%	39.75 ± 5.45%	62.78 ± 6.50%
Pearson's correlation coefficient	RαPhogrin:MαPhogrin	0.84 ± 0.01			
	ChromograninB:GAD67	n/a	n/a	0.12 ± 0.02	
		**SOMAS**		**PUNCTA**	
	Phogrin:ChromograninB	0.04 ± 0.04		0.05 ± 0.03	
	Phogrin:VAMP2	0.70 ± 0.03		0.63 ± 0.02	

Using a combination of electron microscopy, immunohistofluorescence and transgenic mice, we have shown that chromogranin B, a canonical LDCV marker, is not expressed in GAD67^+^ neurons in the hippocampus (Figures 1B–D). Surprisingly, phogrin a receptor-type protein tyrosine phosphatase which has been extensively studied in endocrine cells (Wasmeier and Hutton, 1996; Wasmeier et al., 2002, 2005; Torii et al., 2005, 2009) was found highly expressed in specific subtypes of hippocampal interneurons (Figures 4–8). Although, phogrin in the mammalian CNS has been previously reported (Chiang and Flanagan, 1996), its neuronal expression pattern and subcellular distribution have been only tangentially addressed. To the best of our knowledge this is the first report to identify phogrin expression in hippocampal interneurons. Furthermore, our findings clearly demonstrate that phogrin is highly expressed in PV^+^ interneurons throughout different hippocampal regions. These findings are consistent with the presence of phogrin mRNA in the primordial Medial Ganglionic Eminence (MGE) (Chiang and Flanagan, 1996) that supplies the vast majority of PV^+^ hippocampal and cortical interneurons (Tricoire et al., 2011). Intriguingly, our preliminary data suggest that phogrin colocalizes with PV^+^ cells exclusively in the hippocampus (Supplemental Figure 5). These results indicate that although their common origin, PV^+^ interneurons may exhibit different molecular content in a region-specific manner that may be related to distinct functionality. Furthermore, phogrin expression was tightly confined to a specific subset of interneurons since SOM^+^ and CCK^+^ cells, other prominent basket cell types in the hippocampus, lack phogrin expression altogether (Figures 4, 5). These results are in contrast with previous studies in neurosecretory cells of the gastrointestinal tract, in which phogrin can be found in both SOM^+^ and CCK^+^ cells (Gomi et al., 2013) suggesting that phogrin expression is regulated in a tissue-specific manner.

Consistent with molecularly distinct pools of secretory vesicles, phogrin and chromogranin B, appeared markedly segregated in hippocampal neurons, with chromogranin B mainly confined to excitatory CA3 and DG neurons (Figures 1, 2). This segregated expression may not be exclusive to the mouse hippocampus as insulin-containing vesicles in pancreatic β-cells mostly contain phogrin with just a small fraction of vesicles with detectable chromogranin B (Giordano et al., 2008). These data indicate that different neuronal populations display distinct LDCVs with non-overlapping molecular markers. In interpreting these results it is important to consider that while our immunohistofluorescence approach allows us to assess endogenous phogrin without the confounding of overexpression, it is limited by antibody specificity and sensitivity. In order to overcome these limitations, we have used two antibodies to confirm the expression of phogrin in hippocampal interneurons (Supplemental Figure 1). Antibody characterization confirms that both a commercially available antibody and an antibody generated for electron microscopy experiments by Torii et al. exhibit indistinguishable staining patterns (Supplemental Figure 1). Even so, we cannot rule out the presence of phogrin, although to a lesser extent, in excitatory neurons. However, the absence of chromogranin B in PV^+^ interneurons is strengthened by the undetectable levels of this protein in GAD67^+^ cells. Within inhibitory neurons, phogrin-containing vesicles exhibit a ubiquitous expression in both axon terminals and somato-dendritic compartments. Double immunohistofluorescence of VAMP2 and phogrin showed high colocalization levels in regions showing a punctate pattern of staining (Figures 3B,C) according to phogrin immunoparticles at axon terminals (Figure 3A). Additionally, a significant proportion of immunoparticles for phogrin were found at postsynaptic sites in dendritic shafts of interneurons suggesting that phogrin-containing vesicles may be stored at both presynaptic and postsynaptic regions. A postsynaptic location of phogrin is not surprising since somato-dendritic exocytosis has been described for many neuromodulators (Fried et al., 1985; Zhu et al., 1986; van den Pol, 2012).

A pressing question is the function of phogrin in regulating vesicle dynamics in hippocampal interneurons. Previous studies in neuroendocrine cells and neurons proposed diverse roles of phogrin in LDCVs trafficking (Wasmeier et al., 2002, 2005; Saito et al., 2011), exocytosis (Cai et al., 2004), endocytosis (Torii et al., 2005; Wasmeier et al., 2005), and cargo loading (Henquin et al., 2008). Our data indicates phogrin may regulate distinctive functions depending on neuronal type and cargo content. Furthermore, taken together the observed cellular specificity of phogrin and chromogranin B challenges the classic view of secretory vesicles as molecularly homogenous vesicles. According to this, distinct modes of LDCVs exocytosis, transient and persistent, have been described in cortical and hippocampal neurons (de Wit et al., 2009; Farina et al., 2015) implying the existence of different LDCVs pools in a similar fashion to small synaptic vesicles. In addition to the molecular composition, the balance between transient and persistent release may also be determined by LDCVs cargo (de Wit et al., 2009).

Interestingly, we have found that a vast majority of phogrin^+^ cells in CA3 and DG neurons colocalized with NPY and that most of these neurons were also PV^+^ in a region-specific manner (Figures 7, 8). A potential explanation for the region specificity is that since both phogrin and PV expression is fairly homogenous throughout the different hippocampal subregions, the increase in the colocalization ratio may be likely reflecting the higher levels of NPY expression in the DG, which is well-characterized (Deller and Leranth, 1990; Gruber et al., 1994). In this scenario, the higher ratio of colocalization in CA3 and DG neurons may just be reflecting the higher probability of NPY expression in these cells. Nonetheless, increasing numbers of NPY^+^ and PV^+^ interneurons is expected to have an impact in the regulation of excitatory synaptic transmission and information processing in these hippocampal subregions. Thus, NPY in the DG has been proposed to be an important factor in the modulation of epilepsy (Erickson et al., 1996; Klapstein and Colmers, 1997; Gariboldi et al., 1998). Given the high levels of colocalization of phogrin with NPY, it would be interesting to address the potential implication of this protein in regulating NPY levels and its relationship to epileptic seizures. An interesting hypothesis would be that phogrin mediates NPY secretion in the hilar hippocampal region. In this scenario, phogrin loss of function may lead to aberrant hyperexcitability due to decreased levels of NPY. Therefore, cargo-specificity may be related with phogrin function in the adult hippocampus in the same manner as has been shown in neurosecretory cells (Henquin et al., 2008). Undoubtedly, further experiments will be necessary to elucidate the role of phogrin in hippocampal neurons and secretory vesicle dynamics. Nonetheless, the identification of phogrin as a specific marker of vesicles in inhibitory cells in the mouse hippocampus will be a highly valuable new tool for the study of the role of interneuronal neuropeptide secretion in regulating neuronal networks.

## Author contributions

JR, FM, and SJ designed the work. JR performed the experiments and analyzed the data. RL performed electron microscopy experiments. JR, FM, and SJ wrote the manuscript.

## Funding

NARSAD (Ref 22688) Young Investigator Award to SJ. Spanish Ministry of Education and Science (BFU2015-63769-R) and Junta de Comunidades de Castilla-La Mancha (PPII-2014-005-P) to RL.

### Conflict of interest statement

The authors declare that the research was conducted in the absence of any commercial or financial relationships that could be construed as a potential conflict of interest.
