# Supplementary figures and images for: Excitatory and Inhibitory Neurons in the Hippocampus Exhibit Molecularly Distinct Large Dense Core Vesicles
Source: Front Cell Neurosci. 2016 Aug 31;10:202. doi: 10.3389/fncel.2016.00202 (PMC5005380; doi:10.3389/fncel.2016.00202)

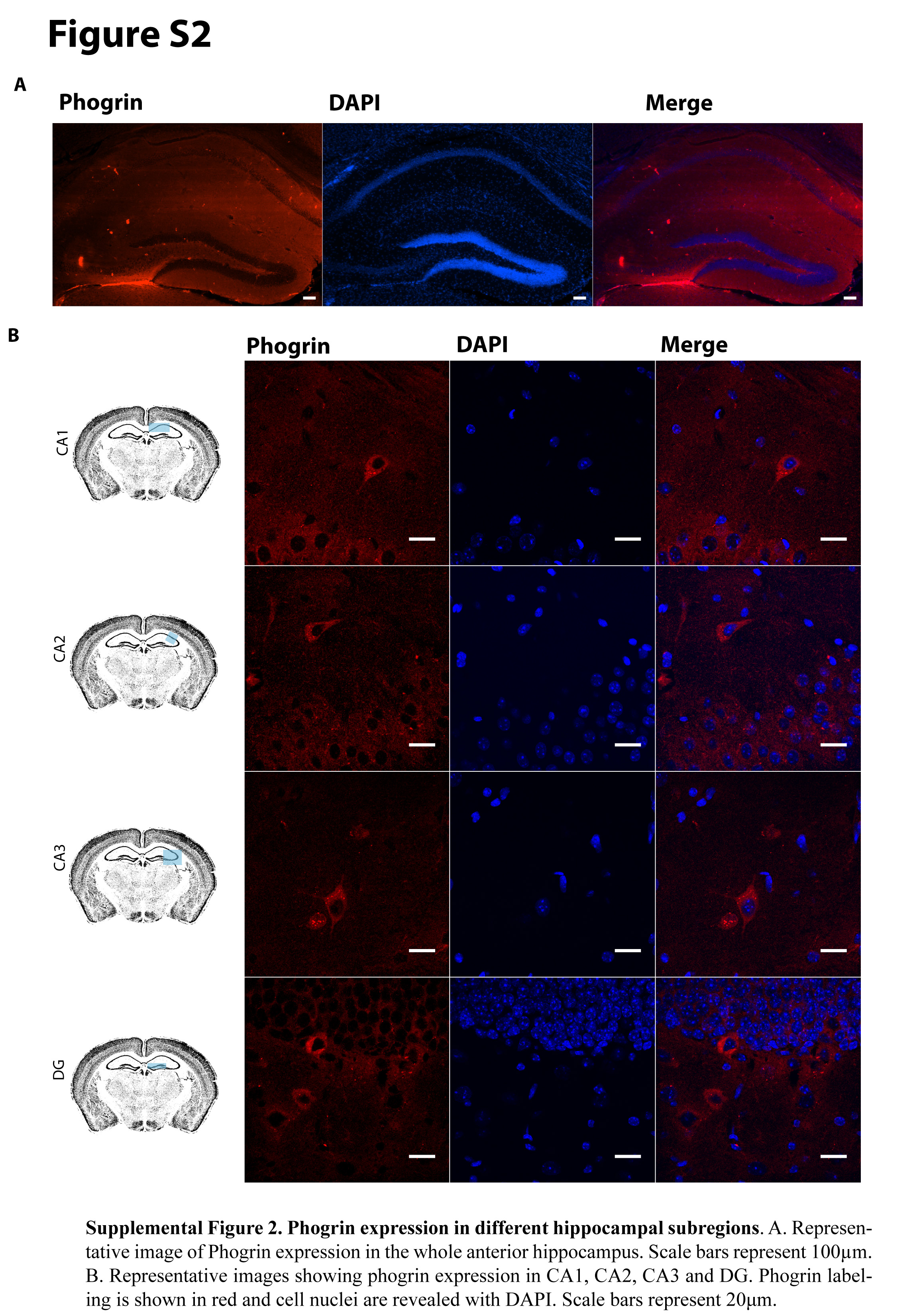

Supplement: Supplementary file 1 [file Presentation1.zip › New folder/Supplemental Figure 2.jpeg]

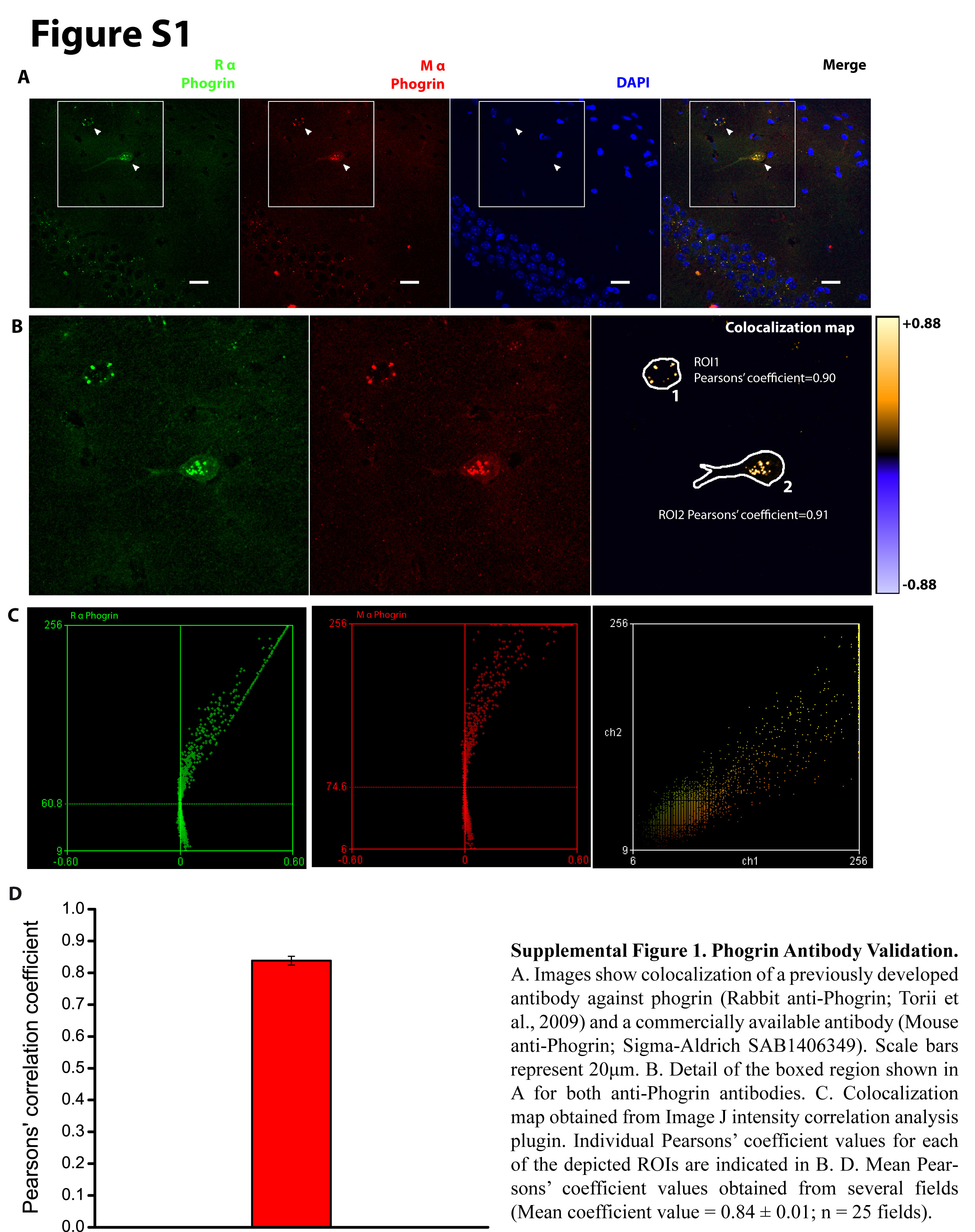

Supplement: Supplementary file 1 [file Presentation1.zip › New folder/Supplemental Figure 1.jpeg]

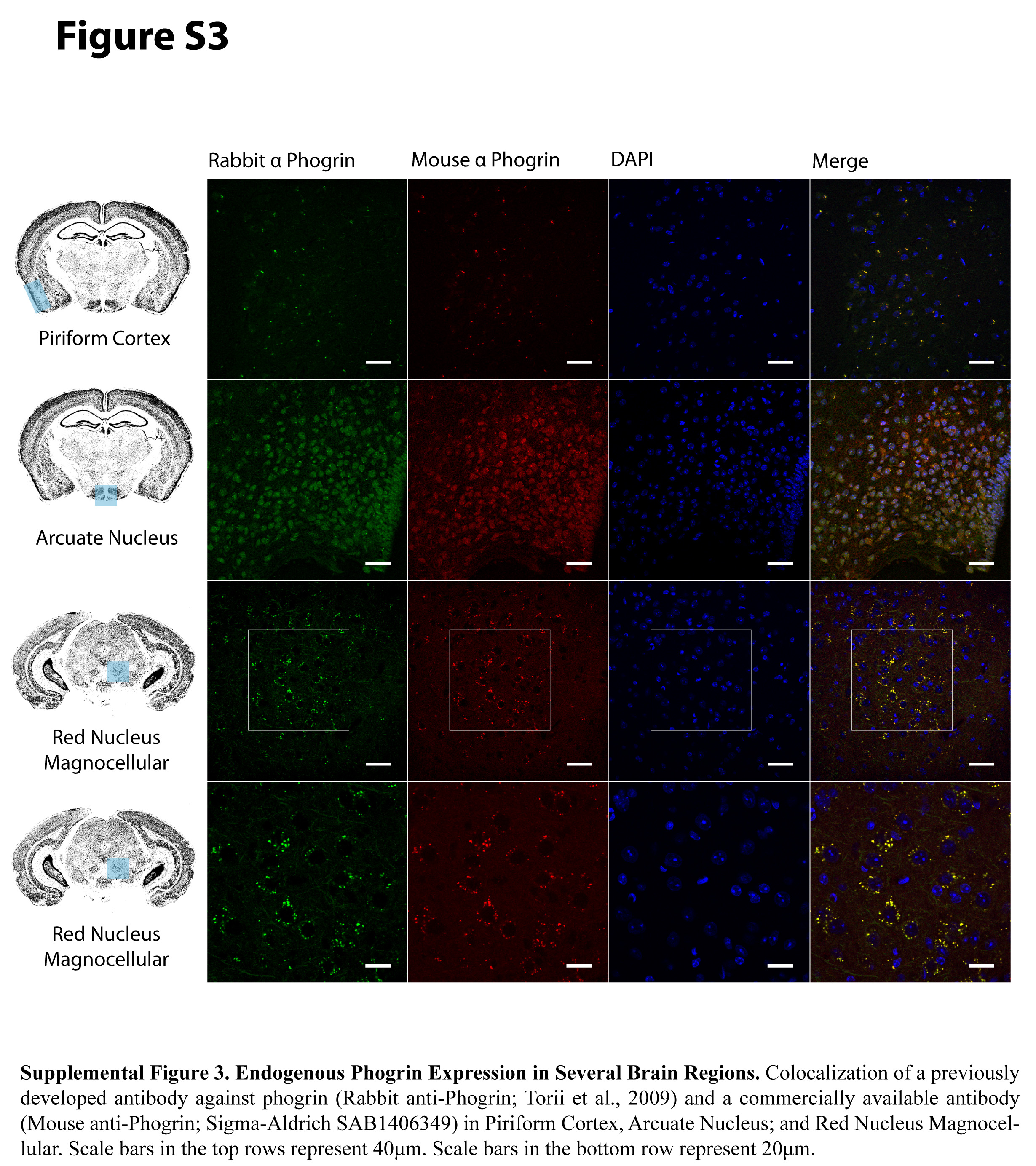

Supplement: Supplementary file 1 [file Presentation1.zip › New folder/Supplemental Figure 3.JPEG]

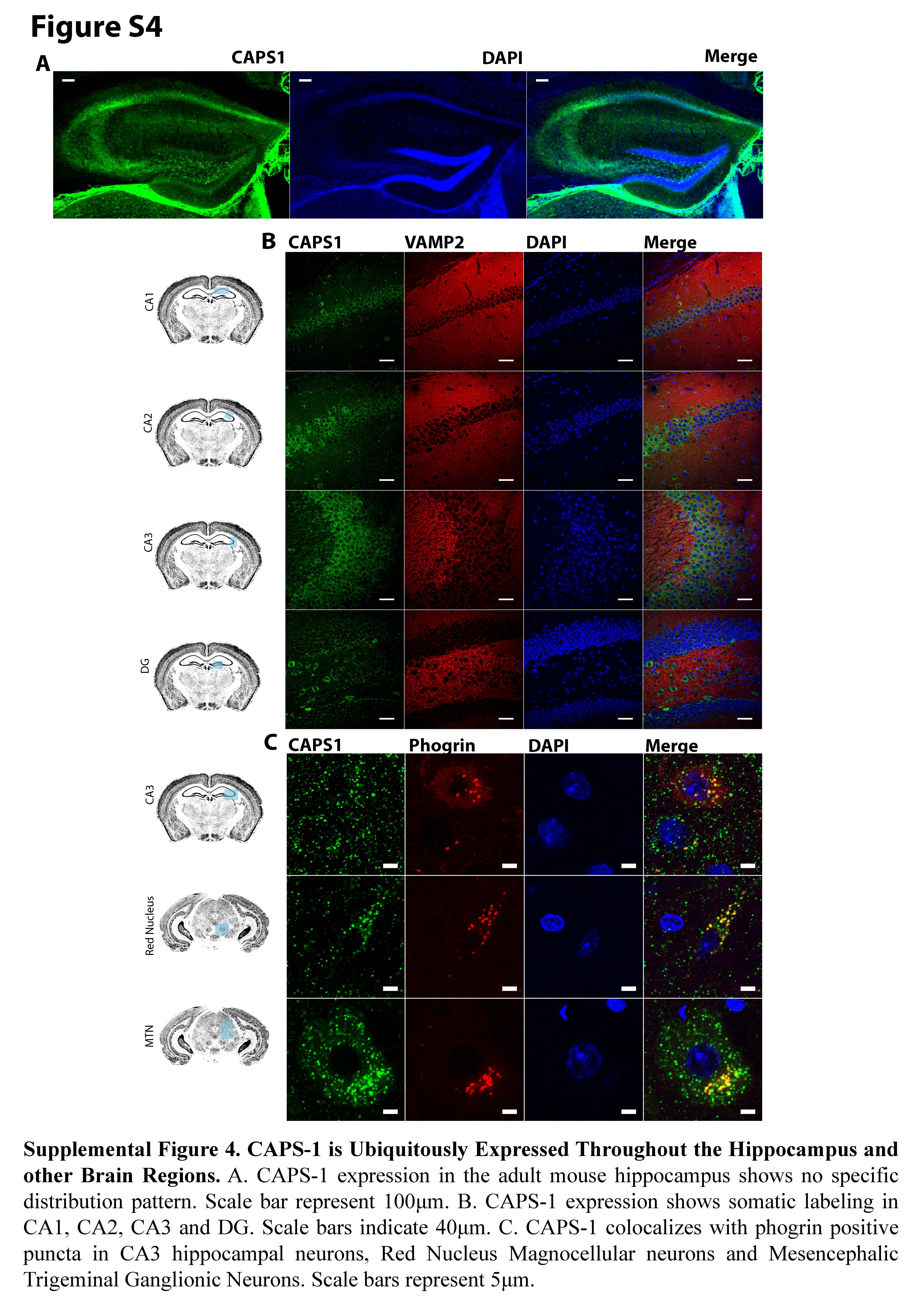

Supplement: Supplementary file 1 [file Presentation1.zip › New folder/Supplemental Figure 4.jpg]

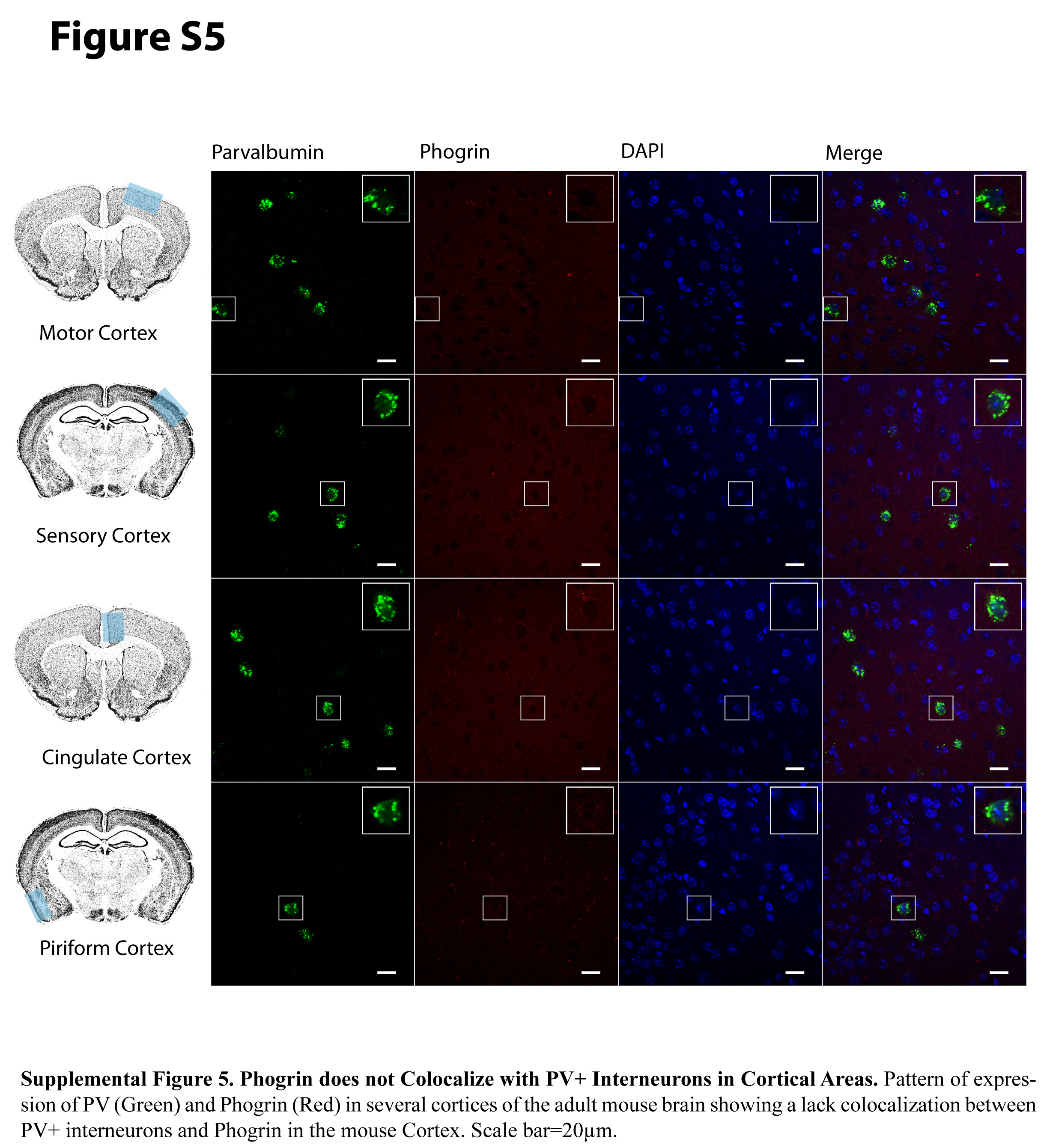

Supplement: Supplementary file 1 [file Presentation1.zip › New folder/Supplemental Figure 5.jpeg]
